# Ohio Medical College, and Surgical Journal

**Published:** 1852-07

**Authors:** 


*The Ohio Medical and Surgical Journal.*—The May No. of this excellent journal is before us, and as usual, contains much which will be interesting to the profession. Among the original communications we see reported cases of Rheumatic Inflammation, by L. Mendenhall, M. D. Although not engaged in general practice, we have seen within the last few years, several cases which lead us to regard the views of Dr. Mendenhall, as correct.

We notice, also, an interesting, because singular, case of stricture of the Rectum, by John N. Beach, M. D. It is rather remarkable that the patient should have lingered for fifty days in this condition.

The profession in the interior of the State, appear to contribute with some spirit to this journal. It is edited by Richard L. Howard, M. D., Professor of Surgery in Starling Medical College, is published Bi-monthly each No. containing ninety-six pages, at the low price of two dollars per annum.

